# Effect of hospital volume on gastric cancer resection outcome in Switzerland: 24-year nationwide retrospective analysis

**DOI:** 10.1093/bjsopen/zraf157

**Published:** 2026-01-07

**Authors:** Joël L Gerber, Martin Müller, Martin D Berger, Yves M Borbély, Daniel Candinas, Dino Kröll

**Affiliations:** Department of Visceral Surgery and Medicine, Inselspital, Bern University Hospital, University of Bern, Bern, Switzerland; Department of Emergency Medicine, Inselspital, Bern University Hospital, University of Bern, Bern, Switzerland; Department of Medical Oncology, Inselspital, Bern University Hospital, University of Bern, Bern, Switzerland; Department of Visceral Surgery and Medicine, Inselspital, Bern University Hospital, University of Bern, Bern, Switzerland; Department of Visceral Surgery and Medicine, Inselspital, Bern University Hospital, University of Bern, Bern, Switzerland; Department of Visceral Surgery and Medicine, Inselspital, Bern University Hospital, University of Bern, Bern, Switzerland

**Keywords:** gastrectomy, postoperative complications, morbidity, quality indicators, caseload, patient safety

## Abstract

**Background:**

Postoperative mortality is a key indicator of surgical quality and central to volume–outcome research, which has shaped minimum case volume standards. In Switzerland, evidence for gastric cancer surgery outcomes remains limited, and regulation is still debated. This study analysed nationwide perioperative volume–outcome associations.

**Methods:**

The study comprised an analysis of the inpatient database from the Swiss Federal Statistical Office. Patients undergoing surgical or endoscopic resection for gastric cancer between 1998 and 2021 were included. Data were stratified by surgical caseload (quartiles), hospital inpatient volume, and hospital type. Outcomes included in-hospital mortality, failure to rescue, and perioperative morbidity.

**Results:**

Some 8708 patients from over 30 million hospital admissions were included. The annual resection volume increased from 290 in 2000 to 432 in 2020. The overall in-hospital mortality rate was 3.9%, with an inverse association with surgical caseload (2.2% in centres performing > 20 resections annually *versus* 2.8, 4.2, and 4.6% in lower-volume quartiles; *P* = 0.001). Similar correlations were observed for hospitals with > 35 000 inpatient admissions annually (2.3 *versus* 3.6 and 4.7%; *P* < 0.001) and for university hospitals (2.0 *versus* 4.2 and 4.3%; *P* < 0.001). Although the reported proportion of severe complications was higher, the rate of failure to rescue was lower in hospitals with high inpatient volumes (*P* < 0.001) and in university hospitals (*P* = 0.002).

**Conclusion:**

The findings of lower rates of in-hospital mortality and failure to rescue in hospitals with higher surgical and inpatient volumes support the potential value of centralization in gastric cancer surgery, and may guide future discussions on regulation.

## Teaser

Nationwide Swiss data over 24 years revealed strong perioperative volume–outcome relationships in gastric cancer resections. Outcomes improved not only with higher surgical caseloads but also with greater hospital inpatient volumes, broadening the focus from procedural metrics to hospitalwide, interdisciplinary care. These population-level data support centralization and enhance the evidence base for ongoing regulatory discussions.

## Introduction

The correlation between treatment volume and clinical outcomes has led to the implementation of minimum case volume regulations in several countries across a range of disciplines. These measures aim to improve patient safety and quality of care, particularly for complex and rare treatments. In Switzerland, the framework of highly specialized medicine (HSM)^1^ governs the allocation of complex, resource-intensive, and low-frequency treatments to hospitals. The objective is to centralize such services in institutions with the necessary expertise, infrastructure, and procedural volume to ensure high-quality and cost-effective medical care^1^.

Within the domain of gastrointestinal surgery, the HSM framework regulates oesophageal, liver, pancreatic, and low rectal resections, as well as complex bariatric surgery^2^. These procedures are recognized for their technical demands and the need for multidisciplinary management. Although gastric cancer resections are relatively rare, complex, resource-intensive, bear high innovation potential, and require multidisciplinary care, they have not yet been subjected to similar regulation in Switzerland^2^. This becomes particularly relevant if recently proposed benchmarks for partial and total gastrectomy are to be achieved^3^. This regulatory gap is of note considering Switzerland’s position in European cancer survival statistics: between 2010 and 2014, Switzerland achieved the highest 5-year survival rates for oesophageal cancer, while ranking in the upper–mid range for overall gastric cancer survival across Europe^4,5^.

There is evidence to support the role of treatment volume in influencing outcomes for patients with gastric cancer. In Europe, studies from the UK^6^, Germany^7^, Italy^8,9^, Spain^10^, France^11^, and the Netherlands^12^ have demonstrated a relationship between higher annual surgical volume and reduced in-hospital mortality. Furthermore, studies from different countries^13–16^ have also suggested that hospital type may influence surgical outcomes. Still, the minimum regulations for gastric cancer resections vary significantly in Europe. Countries such as the Netherlands^17^ and Denmark^18^ have implemented formal centralization strategies, whereas others rely on recommendations from scientific societies or regional initiatives. Data from Switzerland examining these relationships in the context of gastric cancer remain limited, hindering informed debate on potential regulation.

The present study aimed to fill this gap by analysing perioperative volume–outcome relationships for oncological gastric resections in Switzerland between 1998 and 2021 using a nationwide inpatient database. Associations between annual surgical volume, annual hospitalwide inpatient volume, and hospital type and the outcomes in-hospital mortality, failure to rescue (FTR), and perioperative morbidity were explored. The findings may guide future regulatory decisions and contribute to the internationally ongoing discourse on centralization of gastric cancer resections.

## Methods

### Study design

This analysis of a national inpatient database, named Medical Statistics of Hospitals, and provided by the Swiss Federal Statistical Office (FSO), covered the years 1998–2021^19^. The database comprises all hospital admissions in Switzerland that are reported mandatorily each year. Each hospital is assigned a unique anonymized identifier (hospital ID) per year. Mandatory reporting includes sociodemographic (for example age, sex), administrative (such as hospital type, patient insurance class, admission source), and clinical information (for example diagnoses, treatments, medications). Diagnoses are coded according to the International Classification of Diseases, 10th Revision (ICD-10). Diagnostic and surgical procedures are recorded using the Swiss Classification of Operations (CHOP), which is an adapted edition of the American classification system (ICD-9-CM, volume 3). When patients are transferred, each hospital reports a hospital admission, whereas procedures are documented exclusively by the hospital in which they were performed.

This study is based on the aforementioned anonymized data, which are provided for research purposes under a data use agreement. In accordance with the Swiss Human Research Act (SR 810.30), studies using fully anonymized data are exempt from ethical committee review.

### Eligibility criteria

Patients aged ≥ 16 years were included if they had gastric cancer (ICD-10, C16) as a main diagnosis, and underwent surgical or endoscopic gastric resection coded as the main procedure (*Table S1*). Patients aged < 16 years and those with a different primary diagnosis or main treatment were excluded.

### Outcomes of interest

Main outcomes were in-hospital mortality and FTR. Secondary outcomes included reinterventions (revisional surgery, postoperative endoscopy), as well as intensive care treatment, mechanical ventilation, and readmissions as dichotomous variables.

### Data extraction

The following variables were extracted: patient age and sex, hospital ID and type, primary and secondary diagnoses, primary and secondary procedures with dates, and date of death.

### Definitions

Hospital type was defined according to the FSO as university (tertiary care), centrum (secondary care), and other (primary care). The classification itself is based on the cumulative weighted score of medical specialty training categories (university > 100; centrum 20–100; other < 20), and annual inpatient volume (university > 35 000; centrum 9000–35 000; other < 9000)^20^. One of these two criteria must be fulfilled for a hospital to be classified as non-academic centrum, whereas university hospitals are classified as such by default^20^. An index hospital admission was defined as the primary admission with gastric cancer as the main diagnosis and gastric resection as the main treatment. In-hospital mortality was defined as death during the index hospital admission within 30 days of the primary gastric resection. FTR was defined at the hospital level as in-hospital mortality among patients with severe complications. Severe complications were defined as shown in *Table S2*, adapted from previous literature^21,22^ and the availability of extractable variables. Surpassing FTR was defined as the observed number of FTR cases minus the expected optimum number, calculated by applying the lowest observed FTR rate within each stratification group as reference. Revisional surgery and postoperative endoscopy were defined as a secondary procedure between 1 and 30 days after the primary resection.

### Statistical analysis

Data were stratified by: hospital type; annual gastric cancer surgery volume quartiles, using the past 5 years (2017–2021) as benchmark; and overall annual hospital inpatient volume. Charlson Co-morbidity Index (CCI) score was calculated from secondary diagnoses using the Stata module CHARLSON^23^. The Kruskal–Wallis test was used for group comparisons (> 2 groups) of continuous variables, which are described using median (interquartile range). Categorical variables are shown as numbers with percentages, and were compared between groups using the χ^2^ test of independence. *P* < 0.050 was considered statistically significant. Multivariable logistic regression analysis was undertaken to identify factors associated with in-hospital mortality.

Associations between in-hospital mortality and FTR and the continuous exposures (surgical and inpatient volume) were analysed using restricted cubic spline regression within a mixed-effects generalized linear model. The model included a random intercept for hospital ID, assumed a Bernoulli distribution, and used a logit link function. Adjustments were made for patient-level confounders known to predict in-hospital mortality, including sex, age, and co-morbidities (CCI). The optimal number of spline knots (3–7) was selected by minimizing the Akaike information criterion. Statistical analyses were carried out using Stata^®^ MP version 16.0 (StataCorp, College Station, TX, USA). Plots were generated using Python version 3.13.0 (Python Software Foundation, Wilmington, DE, USA).

## Results

**Fig. 1:**
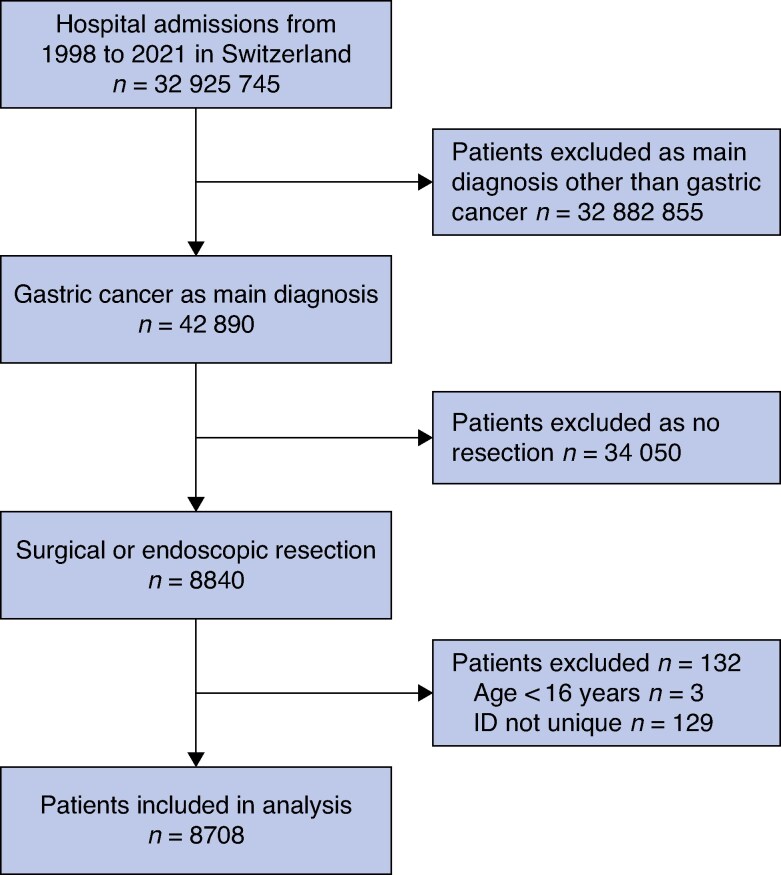
Study flow chart ID, patient identifier.

**Fig. 2:**
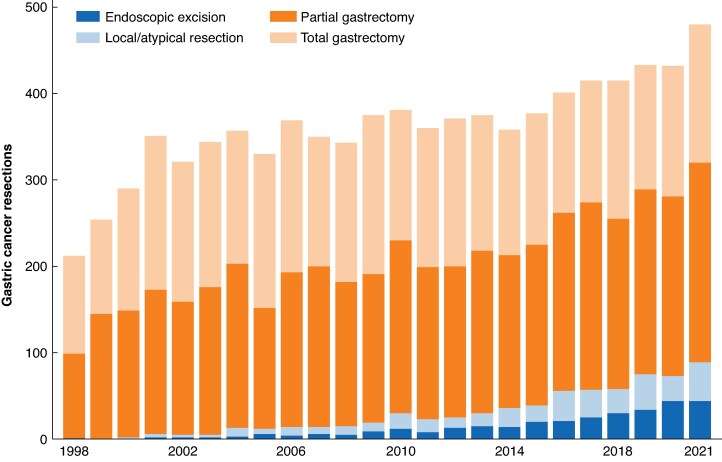
Temporal trends in gastric cancer resection volumes

A total of 8708 gastric cancer resections were identified from over 30 million hospital admissions between 1998 and 2021 (*Fig. 1*). The development of gastric resections over time is shown in *Fig. 2*. The number of gastric cancer resections grew progressively over time (290 in the year 2000, 381 in 2010, and 432 in 2020). This rise was driven to a similar extent by endoscopic and local/atypical surgical resections on the one hand, and partial gastrectomies on the other. The proportion of endoscopic and local resections increased from 0.7% in 2000 to 16.9% in 2020.

### Clinical characteristics

Centres were stratified by annual gastric cancer surgery volume (quartiles), annual hospital inpatient volume, or hospital type for evaluation of volume–outcome relationships. Based on data from the final 5 years (2017–2021), surgical volume quartiles corresponded to < 7, 7–13, 14–20, and > 20 resections annually. Distributions of patient caseloads and intervention types across aforementioned stratification groups are summarized in *Tables S3* and *S4*, respectively. The majority of patients underwent resection in centres of low surgical volume (< 13 yearly resections, 70.9% of patients), intermediate inpatient volume (9000–35 000 annual admissions, 54.2%), and secondary care (54.6%). Centres with high surgical and inpatient volumes, as well as university hospitals, were associated with more frequent use of endoscopic resections (7.8–10.0%). In contrast, local/atypical resections appeared to be relatively more frequent in mid-volume centres, and partial gastrectomies in low-volume centres (*Table S4*). The average surgical volume per centre increased over time in university hospitals, but no such trend was observed in centrum or primary-care hospitals (*Table S5*). Instead, the number of primary-care hospitals performing gastric cancer resections declined, whereas the number of centrum hospitals increased.

Patient characteristics are summarized in *Table S6*. Patients treated in primary-care and low-volume hospitals were older but had a lower co-morbidity burden. Hospital admissions overlapping 2 calendar years accounted for only 29 of the 8708 patients (0.3%).

### In-hospital mortality

**Fig. 3:**
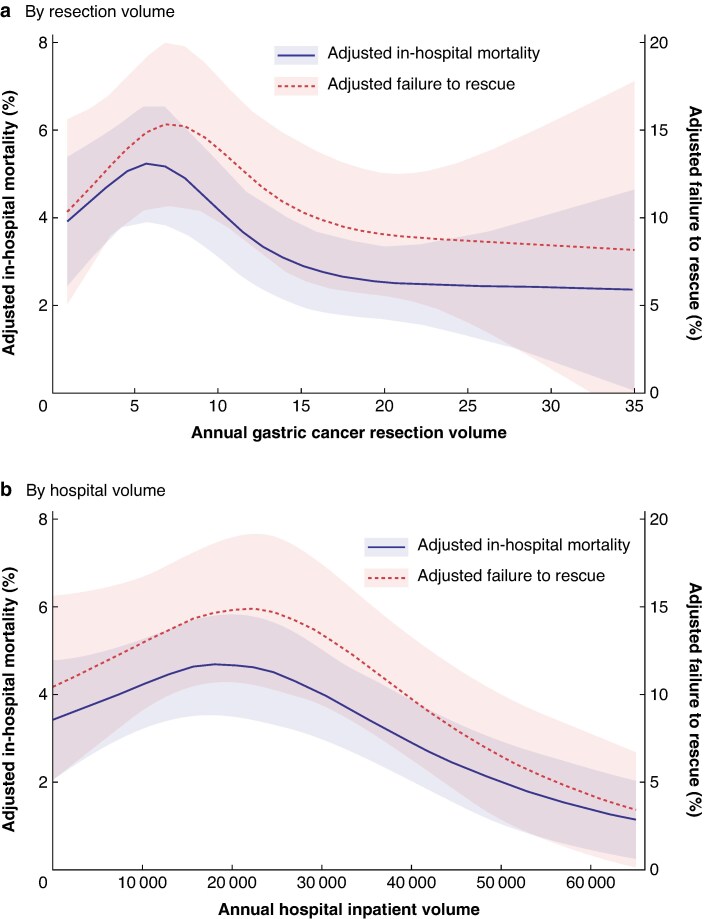
Adjusted volume–outcome relationships Adjusted rates of in-hospital mortality and failure to rescue by **a** annual gastric resection volume and **b** annual hospital inpatient volume. Shaded areas represent 95% confidence intervals.

The overall in-hospital mortality rate was 3.9%, with a slight downward trend across the study period (*Fig. S1*). Higher surgical and inpatient volume as well as hospital type were significantly associated with reduced in-hospital mortality (*Table 1*). The proportion was 2.2% in centres performing more than 20 gastric cancer resections annually, increasing to 4.6% in the lowest quartile (*P* = 0.001). Hospitals with > 35 000 yearly admissions per year reported a rate of 2.3%, whereas proportions ranged from 3.6 to 4.7% in those with ≤ 35 000 (*P* < 0.001). University hospitals reported the lowest inpatient mortality rate at 2.0%, compared with 4.2–4.3% in centrum and primary-care hospitals (*P* < 0.001). Considering only the final decade of the study, differences remained statistically significant for resection volume (*P* = 0.040), whereas trends towards statistical significance were observed for inpatient volume (*P* = 0.051) and hospital type (*P* = 0.065). *Figure 3* shows in-hospital mortality rates by surgical and hospital volume, which were adjusted for age, sex, and co-morbidities to account for heterogeneity in patient characteristics (*Table S7.1–3*).

**Table 1:** In-hospital mortality and failure to rescue

	In-hospital mortality (*n* = 339)	Severe complications (*n* = 1815)	Failure to rescue (*n* = 233)	Surpassing in-hospital mortality	Surpassing failure to rescue
**Annual resection volume**					
> 20 (*n* = 942)	21 (2.2%)	226 (24.0%)	19 (8.4%)	Reference	Reference
14–20 (*n* = 1588)	44 (2.8%)	360 (22.7%)	38 (10.6%)	9 (19.5%)	8 (20.4%)
7–13 (*n* = 2858)	121 (4.2%)	617 (21.6%)	91 (14.7%)	63 (52.4%)	39 (43.0%)
< 7 (*n* = 3320)	153 (4.6%)	612 (18.4%)	85 (13.9%)	79 (51.6%)	34 (39.5%)
P*	0.001	< 0.001	0.167		
**Annual inpatient volume**					
> 35 000 (*n* = 1916)	45 (2.3%)	476 (24.8%)	39 (8.2%)	Reference	Reference
9000–35 000 (*n* = 4721)	220 (4.7%)	1041 (22.1%)	158 (15.2%)	109 (49.6%)	73 (46.0%)
< 9000 (*n* = 2071)	74 (3.6%)	298 (14.4%)	36 (12.1%)	25 (34.3%)	12 (32.2%)
P*	< 0.001	< 0.001	< 0.001		
**Hospital type**					
University (*n* = 1443)	29 (2.0%)	334 (23.1%)	23 (6.9%)	Reference	Reference
Centrum (*n* = 4755)	201 (4.2%)	1088 (22.9%)	151 (13.9%)	105 (52.5%)	76 (50.4%)
Primary care (*n* = 2510)	109 (4.3%)	393 (15.7%)	59 (15.0%)	53 (48.2%)	32 (54.1%)
P*	< 0.001	< 0.001	0.002		

Values are *n* (%). Centres were stratified into quartiles regarding annual gastric resection volume using the past 5 years (2017–2021) as benchmark. Annual hospital inpatient volume (overall) and hospital type were categorized following the classification of the Swiss Federal Statistical Office. Failure to rescue was defined as in-hospital death among patients with severe complications, with proportions referring to the subgroup of patients with severe complications. Surpassing in-hospital mortality was defined as the difference between the observed number of deaths and the best case, which was calculated by applying the lowest observed rate within each stratification group as reference. Proportions of surpassing cases refer to the total number of observed cases. Surpassing failure to rescue was calculated analogously. *χ*^2^* test.

A subanalysis excluding all endoscopic resections and restricted to surgical resections only confirmed increased mortality and FTR rates in hospitals with lower annual resection and overall inpatient volumes (*Table S8*). Findings were similar for subgroup analyses according to resection type (total gastrectomy *versus* partial gastrectomy *versus* atypical resection *versus* endoscopic resection) (*Table S9*), or separating elective and emergency admissions (*Table S10.1–2*).

### FTR

Of the 339 in-hospital deaths, 233 (68.6%) were associated with the reporting of a severe complication, and were therefore classified as FTR cases (*Table 1*). Higher inpatient volume (*P* < 0.001) and hospital type (*P* = 0.002), but not higher surgical volume (*P* = 0.167) were significantly associated with FTR, with proportions ranging from 6.6 to 15.1%. Surpassing FTR accounted for an estimated 76–109 patients, that is 33.4–47.7% of observed FTR cases. FTR rates by surgical and hospital volume, adjusted for patient-level confounders age, sex, and co-morbidities, are shown in *Fig. 3*. To account for the hypothetical bias of under-reporting severe complications in mid- and low-volume centres, adjusted severe complications were estimated by applying the highest observed rate within each stratification group. The resulting adjusted FTR rates and adjusted surpassing FTR rates are shown in *Table S8*.

### Secondary outcomes

Reinterventions and secondary outcomes are summarized in *Table 2*. Low-volume centres reported relatively lower rates of revisional surgery, postoperative endoscopy, and hospital readmission. Mid-volume hospitals had relatively higher rates of mechanical ventilation and readmission compared with high-volume centres. The proportion of patients requiring intensive care was significantly lower in academic hospitals or in those with a high inpatient volume (> 35 000 per year), whereas no relevant difference was observed across groups stratified by resection volume.

**Table 2:** Reinterventions and secondary outcomes

	Revisional surgery (*n* = 310)	Postoperative endoscopy (*n* = 65)	ICU stay (*n* = 3924)	Mechanical ventilation (*n* = 636)	Readmission (*n* = 1537)
**Annual resection volume**					
> 20 (*n* = 942)	40 (4.3%)	16 (1.7%)	413 (43.8%)	74 (7.9%)	230 (24.4%)
14–20 (*n* = 1588)	65 (4.1%)	12 (0.8%)	711 (44.8%)	162 (10.2%)	288 (18.1%)
7–13 (*n* = 2858)	128 (4.5%)	22 (0.8%)	1372 (48.0%)	247 (8.6%)	505 (17.7%)
< 7 (*n* = 3320)	77 (2.3%)	15 (0.5%)	1428 (43.0%)	153 (4.6%)	514 (15.5%)
P*	< 0.001	0.001	0.001	< 0.001	< 0.001
**Annual inpatient volume**					
> 35 000 (*n* = 1916)	105 (5.5%)	23 (1.2%)	715 (37.3%)	161 (8.4%)	341 (17.8%)
9000–35 000 (*n* = 4721)	167 (3.5%)	36 (0.8%)	2465 (52.2%)	437 (9.3%)	896 (19.0%)
< 9000 (*n* = 2 071)	38 (1.8%)	6 (0.3%)	744 (35.9%)	38 (1.8%)	300 (14.5%)
P*	< 0.001	0.004	< 0.001	< 0.001	< 0.001
**Hospital type**					
University (*n* = 1443)	69 (4.8%)	18 (1.3%)	392 (27.2%)	96 (6.7%)	254 (17.6%)
Centrum (*n* = 4755)	194 (4.1%)	40 (0.8%)	2518 (53.0%)	472 (9.9%)	938 (19.7%)
Primary care (*n* = 2510)	47 (1.9%)	7 (0.3%)	1014 (40.4%)	68 (2.7%)	345 (13.7%)
P*	< 0.001	0.002	< 0.001	< 0.001	< 0.001

Values are *n* (%). Centres were stratified into quartiles regarding annual gastric resection volume using the past 5 years (2017–2021) as benchmark. Annual hospital inpatient volume (overall) and hospital type were categorized following the classification of the Swiss Federal Statistical Office. Revisional surgery and endoscopy were assessed within 30 days after resection. Readmissions were captured within the month of discharge or the following month. Intensive care unit (ICU) stay and mechanical ventilation were recorded as binary variables. *χ*^2^* test.

## Discussion

This population-based analysis of Swiss inpatient data found a significant association between in-hospital mortality and FTR and annual gastric cancer resection volume, overall hospital inpatient volume, and hospital type over the 24-year study period.

Overall in-hospital mortality was in the upper European mid-range^6–9,11,15,24–30^, and comparable internationally to data from North America^31,32^, Australia^13,14,33^, and Asia^34,35^ during an overlapping time period, whereas thresholds for high *versus* low volume vary considerably among studies. Nonetheless, recent data from Asia suggest room for optimization^36–38^. Despite Switzerland’s consistently high ranking among global healthcare systems^39,40^, around 90% of in-hospital deaths in this study occurred in hospitals with mortality rates exceeding those of high-volume and tertiary centres. Notably, roughly 40% of all deaths fell above the reference level of high-volume centres, indicating potential room for improvement. These findings align with evidence from a recent meta-analysis^41^ of mainly retrospective data that showed a 35% reduction in mortality after oncological gastric resections for patients treated in surgical high-volume centres relative to their low-volume counterparts. National centralization of gastric cancer surgery in the Netherlands^17^ and Denmark^18,42^, for instance, has led to improved perioperative outcomes. The 30-day mortality rate dropped from 6.4 to 4.1% in the Netherlands^17^ and from 8.2 to 2.4% in Denmark^18^ after implementation of regulations (20 yearly resections and restriction to university hospitals, respectively), confirming that regulation may indeed contribute to optimizing outcome.

Beyond that, the present findings are not only consistent with those of previous studies^13–16^ reporting that in-hospital mortality is influenced by hospital type, but further indicate an association with overall annual inpatient volume. Although the evidence backs mostly the volume–outcome relationship regarding procedure-specific yearly resection volume per hospital or surgeon^6,31,34^, the volume–outcome effect for annual surgical caseload appeared to plateau beyond 20 yearly resections in the present data. This contrasts with the association with total inpatient volume, where mortality continued to improve beyond 35 000 annual admissions without evidence of a plateau, extending current understanding of the topic. Therefore, in-hospital mortality in oncological gastric resections may be determined not only by surgical volume, but also by healthcare system factors, hospital infrastructure, organizational aspects, and multidisciplinary coordination. Higher inpatient volumes may reflect improved access to continuous emergency care, advanced endoscopy, and interventional services. Increased rates of local and endoscopic resection may indicate more rapid adoption of innovative, potentially organ-preserving strategies in high-volume and tertiary centres. Such considerations may be particularly relevant in smaller countries or sparsely populated regions.

FTR followed a pattern comparable to that of in-hospital mortality across hospital stratification groups. Between one-third and one-half of such events occurred at rates exceeding those of high-volume or tertiary centres, even after adjusting for patient-level confounders including age, sex, and co-morbidities to account for differences in patient characteristics between subgroups. In line with this, reintervention rates were lower in low-volume and primary-care hospitals, aligning with previous Canadian data^31^. Assuming comparable true complication rates, high-volume settings might hence be associated with a lower threshold for reintervention. This could potentially explain the higher observed rates of severe complications, although the data do not permit differentiation between under-reporting and underdetection as sources of bias.

In this analysis, very low-volume centres exhibited slightly lower adjusted proportions for mortality and FTR compared with low- and mid-volume hospitals, resulting in surpassing mortality and FTR rates that were closer to those of high-volume and tertiary-care hospitals. One explanation may lie in institutional specialization within Swiss healthcare. High-volume hospitals typically rely on dedicated upper gastrointestinal surgeons, whereas very low-volume centres may have a single general surgeon managing the entire perioperative pathway, ensuring consistency. On the other hand, mid-volume centres might involve several general surgeons, lowering individual case volumes and increasing variability. Teaching status could also matter; data from the USA have shown lower 30-day mortality rates in teaching compared with non-teaching hospitals among institutions with < 100 beds^43^. Moreover, differences between public and private hospitals might have influenced outcomes, but are not captured in this data set.

This observational study, based on administrative registry data at a population level, does not allow causal inference and is subject to several inherent limitations. The registry does not include standardized complication grading^21^ and cancer staging. Selection and information bias may have arisen owing to non-random treatment allocation, varying coding accuracy, and reporting of complications across institutions. Moreover, the CHOP classification does not distinguish between laparoscopic and open oncological gastric resections. As laparoscopy (54.21.20 as surgical approach, or 54.21 in general), or laparotomy (54.1) were coded in only a minority of cases simultaneously with gastric surgery, 92.4% of operations were documented without the surgical approach, precluding analysis of the role of minimally invasive surgery. Previous studies^3,38,44^ have partly reported higher morbidity but not increased mortality for open approaches in gastric cancer surgery, leaving the role of minimally invasive surgery undetermined in this analysis. Still, in-house mortality represents an objective endpoint, and procedure coding—being crucial for billing—is typically accurate, thereby minimizing the risk of documentation bias in these outcomes very relevant to regulations. Furthermore, FTR is a valuable indicator for quality measurement both within hospitals and across healthcare systems. Nonetheless, temporal trends and evolving treatment standards over the extended study period may have introduced heterogeneity not fully captured in the analysis. The constraints of observational, registry-based data underscore the importance of complementary prospective research. Further research is needed to examine how surgical and inpatient volume interact with hospital type, with a particular focus on identifying hospital structural conditions and institutional thresholds necessary to achieve favourable outcomes and preserving quality of life.

In conclusion, this nationwide analysis has indicated that perioperative outcomes of gastric cancer resections are influenced not only by surgical caseload, but also by overall inpatient volume and type of hospital institution. Recognizing the role of hospital infrastructure and organizational factors may further support the development of more targeted healthcare policies and quality assurance strategies, both within and beyond the Swiss healthcare system.

## Supplementary Material

zraf157_Supplementary_Data

## Data Availability

The original data set is subject to contractual restrictions imposed by the Swiss FSO and must be deleted by the authors upon publication. It is, however, available from the Swiss FSO upon reasonable request. Any further information is available upon request.
