# Supplementary material for: Effect of hospital volume on gastric cancer resection outcome in Switzerland: 24-year nationwide retrospective analysis
Source: BJS Open. 2026 Jan 7;10(1):zraf157. doi: 10.1093/bjsopen/zraf157 (PMC12777974; doi:10.1093/bjsopen/zraf157)
Supplement: zraf157_Supplementary_Data [file zraf157_supplementary_data.docx]

**Gastric cancer resection outcomes in Switzerland: a 24-year retro-spective analysis of hospital volume, perioperative mortality, and failure to rescue**

Joël L. Gerber^1^, Martin Müller^2^, Martin D. Berger^3^, Yves M. Borbély^1^, Daniel Candinas^1^, Dino Kröll^1^

^1^ Department of Visceral Surgery and Medicine, Inselspital, Bern University Hospital, University of Bern, Switzerland

^2^ Department of Emergency Medicine, Inselspital, Bern University Hospital, University of Bern, Switzerland

^3^ Department of Medical Oncology, Inselspital, Bern University Hospital, University of Bern, Switzerland

**Corresponding author.** Dino Kröll**,** [dino.kroell@insel.ch](mailto:dino.kroell@insel.ch), ORCID [0000-0001-8736-6619](https://orcid.org/0000-0001-8736-6619)

Inselspital, Department of Visceral Surgery and Medicine, Freiburgstrasse, 3010 Bern, Switzerland

**Supplementary Materials - Index**

| **Supplementary Figures and Tables** |  |
| --- | --- |
| **Fig. S1.** In-hospital mortality trend during the study period. | *page 2* |
| **Table S1**. Definition and coding of procedures. | *page 3* |
| **Table S2**. Definition and coding of severe complications. | *page 4* |
| **Table S3**. Patient caseload distribution by stratification groups and subgroups. | *page 5* |
| **Table S4**. Procedure type distribution across stratification groups and subgroups**.** | *page 6* |
| **Table S5.** Centre count and average resection volume by hospital type and year. | *page 7* |
| **Table S6**. Patient characteristics by stratification groups. | *page 8* |
| **Table S7.1.** Factors associated with in-hospital mortality stratified by resection volume. | *page 9* |
| **Table S7.2.** Factors associated with in-hospital mortality stratified by inpatient volume. | *page 10* |
| **Table S7.3.** Factors associated with in-hospital mortality stratified by hospital type. | *page 11* |
| **Table S8.** In-hospital mortality and failure to rescue following surgical resections. | *page 12* |
| **Table S9.** Procedure-related outcomes by stratification groups and sub-groups. | *page 13* |
| **Table S10.1.** In-hospital mortality and failure to rescue for elective admissions. | *page 14* |
| **Table S10.2.** Emergency admission-related outcomes by stratification groups. | *page 15* |
| **Table S11.** Adjusted failure to rescue. | *page 16* |
|  |  |
|  |  |
|  |  |

**Supplementary Figures and Tables**

**Fig. S1.** In-hospital mortality trend during the study period.

**
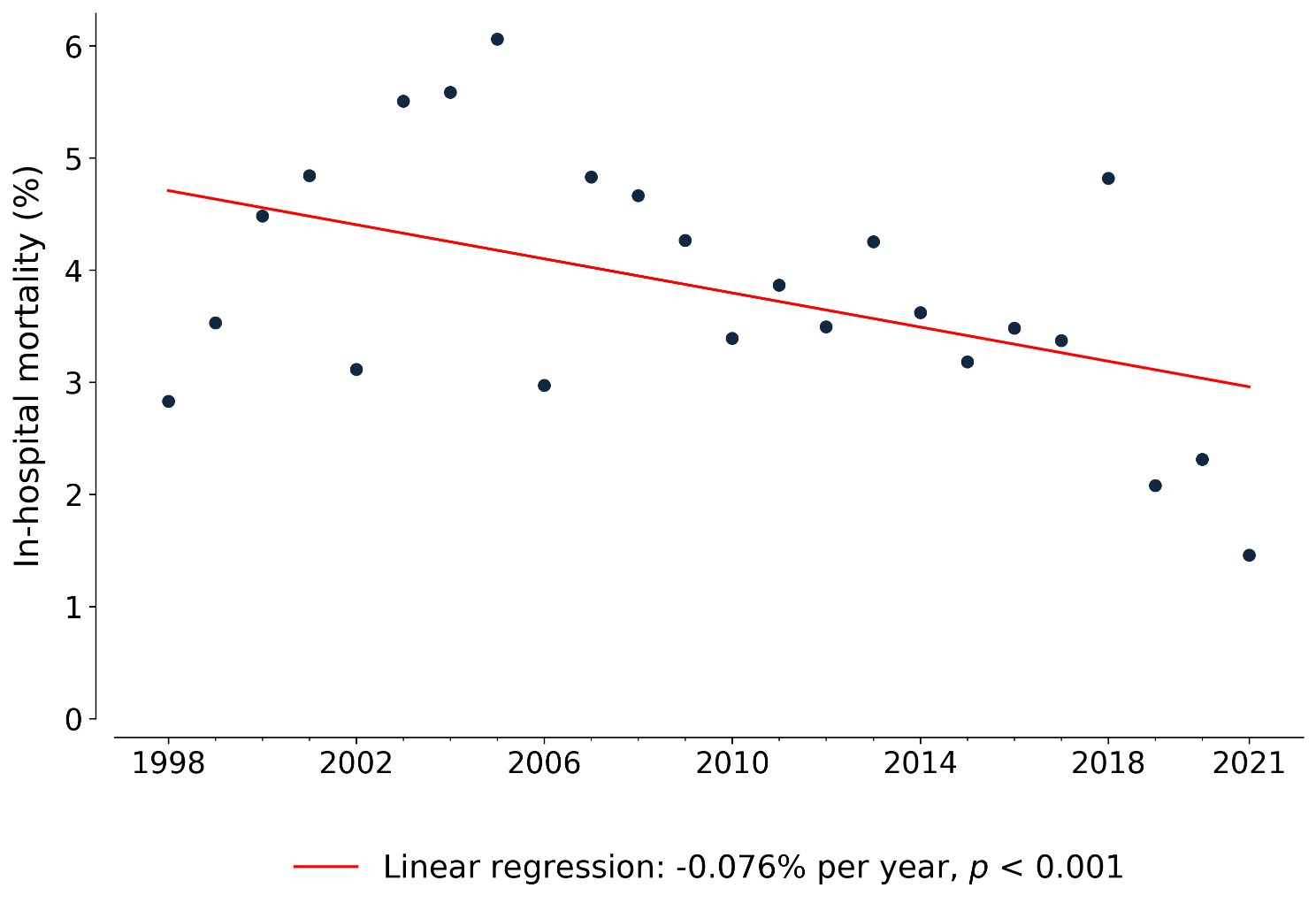
**

*P*-value was derived from linear regression using a t-distribution (degrees of freedom: n - 2).

**Table S1**. Definition and coding of procedures.

| **Procedure type** | **CHOP** |
| --- | --- |
| *Main procedures* |  |
| Total gastrectomy | 43.9 |
| Partial gastrectomy | 43.5, 43.6, 43.7, 43.8 |
| Local/atypical resection | 43.42, 43.49 |
| Endoscopic resection | 43.41 |
| *Secondary procedures* |  |
| Revision surgery | 54.21, 54.1, 54.99, 44.5, 44.40–44.42, 44.49 |
| Postoperative endoscopy | 44.13, 44.43, 44.22 |

Analysis of revision surgery and postoperative endoscopy was restricted to procedures performed between 1 and 30 days after the primary resection. CHOP, Swiss Classification of Operations.

**Table S2**. Definition and coding of severe complications.

| **Severe complications** | **ICD-10** |
| --- | --- |
| *Neurological* |  |
| Coma | R40.2 |
| Acute stroke/cranial haemorrhage | I60–I64 |
| *Cardiovascular* |  |
| Cardiac arrest | I46 |
| Shock, cardiogenic | R57.0 |
| Shock, haemorrhagic | R57.1 |
| Shock, septic | R57.2, R65.21 |
| Shock, postprocedural (unspecified) | T81.1, R57.8, R57.9 |
| Acute myocardial infarction | I21 |
| Pulmonary embolism | I26 |
| Thrombosis of major veins | I80, I81, I82.0, I82.2, I82.3 |
| *Pulmonary* |  |
| Acute respiratory failure | J95.11 |
| Acute respiratory distress syndrome | J80 |
| Pulmonary oedema | J81 |
| Aspiration | J69 |
| Pneumonia | J13–J16, J18, J95.851 |
| *Renal* |  |
| Acute renal failure | N17 |
| *Abdominal* |  |
| Peritonitis | K65 |
| Anastomotic leak | K91.83 |
| Sepsis, postprocedural | R65.20, T81.44, T80.211, A40, A41, R65 |
| *C. difficile* enterocolitis | A04.7 |
| Postoperative ileus | K56.0, K91.3 |
| Acute mesenteric ischemia | K55.0 |
| Gastrointestinal bleeding | K92.0, K92.1, K92.2 |

Surgical site infections (T81.4) were excluded, as the ICD-10 classification does not distinguish between superficial and deep/organ-space infections, and thus does not capture infection severity. ICD-10, International Classification of Diseases, 10th Revision.

**Table S3**. Patient caseload distribution by stratification groups and subgroups.

| **Group** | | **Annual resection volume** | | | | **Annual inpatient volume** | | | **Hospital type** | | |
| --- | --- | --- | --- | --- | --- | --- | --- | --- | --- | --- | --- |
| **Subgroup** | | **< 7** | **7–13** | **14–20** | **> 20** | **< 9k** | **9–35k** | **> 35k** | **PC** | **C** | **U** |
| **Subgroup total** | | **3 320** | **2 858** | **1 588** | **942** | **2 071** | **4 721** | **1 916** | **2 510** | **4 755** | **1 443** |
| **Annual resection volume** | **< 7** | 3 320 | 0 | 0 | 0 | 1 575 | 1 691 | 54 | 2 004 | 1 204 | 112 |
|  | **7–13** | 0 | 2 858 | 0 | 0 | 378 | 2 153 | 327 | 388 | 2 068 | 402 |
|  | **14–20** | 0 | 0 | 1 588 | 0 | 97 | 639 | 852 | 97 | 1 009 | 482 |
|  | **> 20** | 0 | 0 | 0 | 942 | 21 | 238 | 683 | 21 | 474 | 447 |
| **Annual inpatient volume** | **< 9k** | 1 575 | 378 | 97 | 21 | 2 071 | 0 | 0 | 1 886 | 184 | 1 |
|  | **9–35k** | 1 691 | 2 153 | 639 | 238 | 0 | 4 721 | 0 | 624 | 3 942 | 155 |
|  | **> 35k** | 54 | 327 | 852 | 683 | 0 | 0 | 1 916 | 0 | 629 | 1 287 |
| **Hospital type** | **PC** | 2 004 | 388 | 97 | 21 | 1 886 | 624 | 0 | 2 510 | 0 | 0 |
|  | **C** | 1 204 | 2 068 | 1 009 | 474 | 184 | 3 942 | 629 | 0 | 4 755 | 0 |
|  | **U** | 112 | 402 | 482 | 447 | 1 | 155 | 1 287 | 0 | 0 | 1 443 |

Values are *n*. PC, primary care; C, centrum; U, university; k, kilo, i.e. denoting multiplication by one thousand.

**Table S4**. Procedure type distribution across stratification groups and subgroups**.**

| **Resection type** | **Total** | **Partial** | **Local/atypical** | **Endoscopic** |
| --- | --- | --- | --- | --- |
| *n* = 8 078 | 3 706 (42.6) | 4 291 (49.3) | 391 (4.5) | 320 (3.7) |
| **Annual gastric cancer resections** |  |  |  |  |
| >20, *n* = 942 | 385 (40.9) | 417 (44.3) | 46 (4.9) | 94 (10.0) |
| 14–20, *n* =1 588 | 698 (44.0) | 721 (45.4) | 86 (5.4) | 83 (5.2) |
| 7–13, *n* = 2 858 | 1 242 (43.5) | 1 387 (48.5) | 139 (4.9) | 90 (3.1) |
| <7, *n* = 3 320 | 1 381 (41.6) | 1 766 (53.2) | 120 (3.6) | 53 (1.6) |
| *p*-value | 0.207 | < 0.001 | 0.016 | < 0.001 |
| **Annual hospital inpatient volume** |  |  |  |  |
| > 35 000, *n* = 1 916 | 796 (41.5) | 888 (46.3) | 83 (4.3) | 149 (7.8) |
| 9 000–35 000, *n* = 4 721 | 2 072 (43.9) | 2 238 (47.4) | 268 (5.7) | 143 (3.0) |
| < 9 000, *n* = 2 071 | 838 (40.5) | 1 165 (56.3) | 40 (1.9) | 28 (1.4) |
| *p*-value | 0.019 | < 0.001 | < 0.001 | < 0.001 |
| **Hospital type** |  |  |  |  |
| University, *n* = 1 443 | 625 (43.3) | 639 (44.3) | 49 (3.4) | 130 (9.0) |
| Centrum, *n* = 4 755 | 2 046 (43.0) | 2 263 (47.6) | 291 (6.1) | 155 (3.3) |
| Primary care, *n* = 2 510 | 1 035 (41.2) | 1 389 (55.3) | 51 (2.0) | 35 (1.4) |
| *p*-value | 0.278 | < 0.001 | < 0.001 | < 0.001 |

Values are *n* (%). Total, total gastrectomy; partial, partial gastrectomy; local/atypical, local/atypical resection; endoscopic, endoscopic resection. *P*-values were determined using Chi-square test.

**Table S5**. Centre count and average resection volume by hospital type and year.

| **Year** | **University hospitals** | | **Centrum hospitals** | | **Primary care hospitals** | | **In-hospital mortality*** |
| --- | --- | --- | --- | --- | --- | --- | --- |
|  | **Centres** | **Resections per centre** | **Centres** | **Resections per centre** | **Centres** | **Resections per centre** |  |
| 1998 | 2 | 7.5 | 13 | 8.7 | 29 | 2.9 | 2.8 |
| 1999 | 2 | 8.0 | 17 | 5.2 | 50 | 3.0 | 3.5 |
| 2000 | 4 | 8.5 | 19 | 5.1 | 49 | 3.2 | 4.5 |
| 2001 | 5 | 9.4 | 20 | 7.3 | 54 | 2.9 | 4.8 |
| 2002 | 5 | 5.2 | 22 | 5.5 | 63 | 2.8 | 3.1 |
| 2003 | 5 | 10.4 | 22 | 6.1 | 61 | 2.6 | 5.5 |
| 2004 | 5 | 10.6 | 22 | 7.1 | 58 | 2.6 | 5.6 |
| 2005 | 5 | 10.2 | 22 | 5.9 | 55 | 2.7 | 6.1 |
| 2006 | 5 | 9.4 | 24 | 7.5 | 45 | 3.2 | 3.0 |
| 2007 | 5 | 10.6 | 24 | 7.7 | 44 | 2.6 | 4.8 |
| 2008 | 5 | 13.0 | 24 | 6.7 | 42 | 2.8 | 4.7 |
| 2009 | 5 | 11.2 | 23 | 8.8 | 40 | 2.9 | 4.3 |
| 2010 | 5 | 13.0 | 23 | 8.7 | 41 | 2.9 | 3.4 |
| 2011 | 5 | 13.8 | 24 | 7.8 | 32 | 3.3 | 3.9 |
| 2012 | 5 | 14.4 | 24 | 8.8 | 37 | 2.4 | 3.5 |
| 2013 | 5 | 13.0 | 32 | 7.5 | 33 | 2.2 | 4.3 |
| 2014 | 5 | 13.2 | 33 | 7.1 | 25 | 2.4 | 3.6 |
| 2015 | 5 | 14.0 | 32 | 7.6 | 23 | 2.8 | 3.2 |
| 2016 | 5 | 14.0 | 36 | 7.6 | 22 | 2.7 | 3.5 |
| 2017 | 5 | 18.2 | 35 | 7.4 | 21 | 3.1 | 3.4 |
| 2018 | 5 | 16.6 | 37 | 7.6 | 17 | 2.9 | 4.8 |
| 2019 | 5 | 18.6 | 35 | 8.6 | 26 | 1.5 | 2.1 |
| 2020 | 5 | 17.0 | 36 | 7.8 | 19 | 3.5 | 2.3 |
| 2021 | 5 | 19.8 | 35 | 9.6 | 21 | 2.1 | 1.5 |

Values are *n* unless indicated otherwise; *values are %. In-hospital mortality refers to the overall proportion per annum.

**Table S6.** Patient characteristics by stratification groups.

| **Variable** | **Age** | **Female sex*** | **CCI** |
| --- | --- | --- | --- |
|  | **69 (57, 77)** | **3 491 (40.1)** | **3 (2, 8)** |
| **Annual gastric cancer resections** |  |  |  |
| >20 | 65 (55, 75) | 380 (40.3) | 3 (2, 8) |
| 14–20 | 68 (58, 77) | 626 (39.4) | 3 (2, 8) |
| 7–13 | 68 (58, 76) | 1 138 (39.8) | 3 (2, 8) |
| <7 | 70 (60, 78) | 1 347 (40.6) | 2 (2, 8) |
| *p*-value | < 0.001 | 0.866 | < 0.001 |
| **Annual hospital inpatient volume** |  |  |  |
| > 35 000 | 67 (56, 75) | 729 (38.0) | 3 (2, 8) |
| 9 000–35 000 | 69 (58, 77) | 1 926 (40.8) | 3 (2, 8) |
| < 9 000 | 71 (61, 78) | 836 (40.4) | 2 (2, 3) |
| *p*-value | < 0.001 | 0.112 | < 0.001 |
| **Hospital type** |  |  |  |
| University | 65 (54, 74) | 563 (39.0) | 3 (2, 8) |
| Centrum | 69 (59, 77) | 1 903 (40.0) | 3 (2, 8) |
| Primary care | 71 (61, 78) | 1 025 (40.8) | 2 (2, 4) |
| *p*-value | < 0.001 | 0.526 | < 0.001 |

Values are median (i.q.r.) unless indicated otherwise; *values are *n* (%). CCI, Charlson Comorbidity Index. *P*-values were determined using Kruskal-Wallis test for continuous variables, and Chi-squared test for categorical variables.

**Table S7.1.** Factors associated with in-hospital mortality stratified by resection volume.

|  | **Odds ratio** | **95% CI** | ***P*-value** |
| --- | --- | --- | --- |
| **Annual gastric cancer resections** |  |  |  |
| > 20 | 1.00 |  |  |
| 14–20 | 1.20 | 0.70, 2.04 | 0.504 |
| 7–13 | 1.83 | 1.14, 2.94 | 0.013 |
| < 7 | 2.03 | 1.27, 3.25 | 0.003 |
| **Age group** |  |  |  |
| < 45 | 1.00 |  |  |
| 45–59 | 1.68 | 0.58, 4.87 | 0.339 |
| 60–69 | 3.14 | 1.13, 8.73 | 0.028 |
| 70–79 | 6.22 | 2.29, 16.91 | < 0.001 |
| > 80 | 10.88 | 3.99, 29.65 | < 0.001 |
| **Sex** |  |  |  |
| Female | 1.00 |  |  |
| Male | 1.57 | 1.24, 2.00 | < 0.001 |
| **Comorbidities** |  |  |  |
| Charlson Comorbidity Index | 1.14 | 1.10, 1.18 | < 0.001 |

Results of multivariable logistic regression (*n* = 8708). Area under the receiver operating characteristic (AUROC) = 0.729.

**Table S7.2.** Factors associated with in-hospital mortality stratified by inpatient volume.

|  | **Odds ratio** | **95% CI** | ***P*-value** |
| --- | --- | --- | --- |
| **Annual hospital inpatient volume** |  |  |  |
| > 35 000 | 1.00 |  |  |
| 9 000–35 000 | 1.91 | 1.37, 2.65 | < 0.001 |
| < 9 000 | 1.51 | 1.03, 2.21 | 0.037 |
| **Age group** |  |  |  |
| < 45 | 1.00 |  |  |
| 45–59 | 1.68 | 0.58, 4.88 | 0.338 |
| 60–69 | 3.16 | 1.14, 8.77 | 0.027 |
| 70–79 | 6.28 | 2.31, 17.08 | < 0.001 |
| > 80 | 11.15 | 4.09, 30.40 | < 0.001 |
| **Sex** |  |  |  |
| Female | 1.00 |  |  |
| Male | 1.54 | 1.21, 1.96 | 0.001 |
| **Comorbidities** |  |  |  |
| Charlson Comorbidity Index | 1.13 | 1.09, 1.17 | < 0.001 |

Results of multivariable logistic regression (*n* = 8708). Area under the receiver operating characteristic (AUROC) = 0.728.

**Table S7.3.** Factors associated with in-hospital mortality stratified by hospital type.

|  | **Odds ratio** | **95% CI** | ***P*-value** |
| --- | --- | --- | --- |
| **Hospital type** |  |  |  |
| University | 1.00 |  |  |
| Centrum | 1.87 | 1.25, 2.78 | 0.002 |
| Primary care | 2.01 | 1.32, 3.06 | 0.001 |
| **Age group** |  |  |  |
| < 45 | 1.00 |  |  |
| 45 - 59 | 1.65 | 0.57, 4.77 | 0.359 |
| 60 - 69 | 3.04 | 1.09, 8.44 | 0.033 |
| 70 - 79 | 6.00 | 2.21, 16.33 | < 0.001 |
| > 80 | 10.50 | 3.85, 28.62 | < 0.001 |
| **Sex** |  |  |  |
| Female | 1.00 |  |  |
| Male | 1.57 | 1.24, 2.00 | < 0.001 |
| **Comorbidities** |  |  |  |
| Charlson Comorbidity Index | 1.14 | 1.10, 1.17 | < 0.001 |

Results of multivariable logistic regression (*n* = 8708). Area under the receiver operating characteristic (AUROC) = 0.727.

**Table S8.** In-hospital mortality and failure to rescue following surgical resections.

|  | **In-hospital mortality** | **Severe complications** | **Failure to rescue** | **Surpassing in-hospital mortality** | **Surpassing failure to rescue** |
| --- | --- | --- | --- | --- | --- |
|  | ***n* = 336** | ***n* = 1 776** | ***n* = 232** |  |  |
| **Annual resection volume** |  |  |  |  |  |
| >20, *n* = 578 | 12 (2.1) | 122 (21.1) | 10 (8.2) | Reference | Reference |
| 14–20, *n* = 1 615 | 48 (3.0) | 399 (24.7) | 44 (11.0) | 14 (30.1) | 16 (36.5) |
| 7–13, *n* = 2 858 | 121 (4.2) | 626 (21.9) | 92 (14.7) | 67 (55.6) | 45 (51.4) |
| <7, *n* = 3 337 | 155 (4.6) | 629 (18.8) | 86 (13.7) | 86 (55.3) | 28 (32.9) |
| *p*-value | 0.003 | < 0.001 | 0.176 |  |  |
| **Annual inpatient volume** |  |  |  |  |  |
| > 35 000, *n* = 1 767 | 44 (2.5) | 454 (25.7) | 39 (8.6) | Reference | Reference |
| 9 000–35 000, *n* = 4 578 | 218 (4.8) | 1 025 (22.4) | 157 (15.3) | 104 (47.7) | 69 (43.9) |
| < 9 000, *n* = 2 043 | 74 (3.6) | 297 (14.5) | 36 (12.1) | 23 (31.3) | 10 (29.1) |
| *p*-value | < 0.001 | < 0.001 | < 0.001 |  |  |
| **Hospital typology** |  |  |  |  |  |
| University, *n* = 1 313 | 29 (2.2) | 314 (23.9) | 23 (7.3) | Reference | Reference |
| Centrum, *n* = 4 600 | 199 (4.3) | 1 070 (23.3) | 150 (14.1) | 102 (51.1) | 78 (52.3) |
| Primary care, *n* = 2 475 | 108 (4.4) | 392 (15.8) | 59 (15.1) | 55 (50.6) | 29 (48.7) |
| *p*-value | 0.001 | < 0.001 | 0.005 |  |  |

Values are *n* (%). Endoscopic resections were excluded for this subanalysis. Centres were stratified using the annual resection volume quartiles of Table 1, derived from 2017–2021 volumes including endoscopic resections; exclusion of endoscopic cases therefore reclassified some centres to lower quartiles. Annual hospital inpatient volume (overall) and hospital typology were categorized following the classification of the Swiss Federal Statistical Office. Failure to rescue (FTR) was defined as in-hospital mortality among patients with severe complications, with proportions referring to the subgroup of patients with severe complications. Surpassing in-hospital mortality was defined as the difference between the observed number of deaths and the best-case, which was calculated by applying the lowest observed rate within each stratification group as the reference. Proportions of surpassing cases refer to the total number of observed cases. Surpassing failure to rescue was calculated analogously. *P*-values were determined using Chi-squared test.

**Table S9**. Procedure-related outcomes by stratification groups and subgroups.

| **Group** | | **Annual resection volume** | | | | **Annual inpatient volume** | | | **Hospital type** | | |
| --- | --- | --- | --- | --- | --- | --- | --- | --- | --- | --- | --- |
| **Subgroup** | | **< 7** | **7–13** | **14–20** | **> 20** | **< 9k** | **9–35k** | **> 35k** | **PC** | **C** | **U** |
| **Subgroup total,** *n* | | 3 320 | 2 858 | 1 588 | 942 | 2 071 | 4 721 | 1 916 | 2 510 | 4 755 | 1 443 |
| **TG,**  *n* = 3 706 | **IMH** | 84 (6.1) | 63 (5.1) | 22 (3.2) | 13 (3.4) | 41 (4.9) | 118 (5.7) | 23 (2.9) | 55 (5.3) | 111 (5.4) | 16 (2.6) |
|  | **SC** | 265 (19.2) | 302 (24.3) | 179 (25.6) | 115 (29.9) | 133 (15.9) | 491 (23.7) | 237 (29.8) | 173 (16.7) | 521 (25.5) | 167 (26.7) |
|  | **FTR** | 42 (15.8) | 46 (15.2) | 20 (11.2) | 11 (9.6) | 16 (12.0) | 82 (16.7) | 21 (8.9) | 25 (14.5) | 82 (15.7) | 12 (7.2) |
| **PG,**  *n* = 4 291 | **IMH** | 67 (3.8) | 54 (3.9) | 20 (2.8) | 8 (1.9) | 33 (2.8) | 95 (4.2) | 21 (2.4) | 52 (3.7) | 84 (3.7) | 13 (2.0) |
|  | **SC** | 326 (18.5) | 281 (20.3) | 157 (21.8) | 95 (22.8) | 157 (13.5) | 498 (22.3) | 204 (23.0) | 208 (15.0) | 514 (22.7) | 137 (21.4) |
|  | **FTR** | 40 (12.3) | 40 (14.2) | 17 (10.8) | 8 (8.4) | 18 (11.5) | 70 (14.1) | 17 (8.3) | 30 (14.4) | 65 (12.6) | 10 (7.3) |
| **AR,**  *n* = 391 | **IMH** | 1 (0.8) | 4 (2.9) | 0 (0.0) | 0 (0.0) | 0  (0.0) | 5  (1.9) | 0  (0.0) | 1 (2.0) | 4 (1.4) | 0 (0.0) |
|  | **SC** | 17 (14.2) | 25 (18.0) | 8 (9.3) | 6 (13.0) | 7 (17.5) | 36 (13.4) | 13 (15.7) | 11 (21.6) | 35 (12.0) | 10 (20.4) |
|  | **FTR** | 1 (5.9) | 3 (12.0) | 0 (0.0) | 0 (0.0) | 0  (0.0) | 4 (11.1) | 0  (0.0) | 1 (9.1) | 3 (8.6) | 0 (0.0) |
| **ER,**  *n* = 320 | **IMH** | 1 (1.9) | 0 (0.0) | 2 (2.4) | 0 (0.0) | 0  (0.0) | 2  (1.4) | 1  (0.7) | 1 (2.9) | 2 (1.3) | 0 (0.0) |
|  | **SC** | 4 (7.5) | 9 (10.0) | 16 (19.3) | 10 (10.6) | 1  (3.6) | 16 (11.2) | 22 (14.8) | 1 (2.9) | 18 (11.6) | 20 (15.4) |
|  | **FTR** | 0 (0.0) | 0 (0.0) | 1 (6.2) | 0 (0.0) | 0  (0.0) | 1  (6.2) | 0  (0.0) | 0 (0.0) | 1 (5.6) | 0 (0.0) |

Values are *n* (%). TG, total gastrectomy; PG, partial gastrectomy; AR, atypical/local resection; ER, endoscopic resection; IMH, in-hospital mortality; SC, severe complications; FTR, failure to rescue; PC, primary care; C, centrum; U, university; k, kilo, i.e. denoting multiplication by one thousand.

**Table S10.1**. In-hospital mortality and failure to rescue following elective admissions.

Values are *n* (%). Emergency admissions were excluded, leaving only elective cases (*n* = 7655) for this subgroup analysis. *P*-values were determined using Chi-squared test.

|  | **Endoscopic resection** | **In-hospital mortality** | **Severe complications** | **Failure to rescue** |
| --- | --- | --- | --- | --- |
|  | ***n* = 270** | ***n* = 259** | ***n* = 1 422** | ***n* = 176** |
| **Annual resection volume** |  |  |  |  |
| >20, *n* = 853 | 83 (9.7) | 17 (2.0) | 190 (22.3) | 15 (7.9) |
| 14–20, *n* = 1 426 | 73 (5.1) | 36 (2.5) | 284 (19.9) | 32 (11.3) |
| 7–13, *n* = 2 496 | 72 (2.9) | 90 (3.6) | 486 (19.5) | 68 (14.0) |
| <7, *n* = 2 880 | 42 (1.5) | 116 (4.0) | 462 (16.0) | 61 (13.2) |
| *p*-value | < 0.001 | 0.006 | < 0.001 | 0.151 |
| **Annual inpatient volume** |  |  |  |  |
| > 35 000, *n* = 1 697 | 125 (7.4) | 36 (2.1) | 385 (22.7) | 31 (8.1) |
| 9 000–35 000, *n* = 4 152 | 123 (3.0) | 161 (3.9) | 806 (19.4) | 117 (14.5) |
| < 9 000, *n* = 1 806 | 22 (1.2) | 62 (3.4) | 231 (12.8) | 28 (12.1) |
| *p*-value | < 0.001 | 0.003 | < 0.001 | 0.007 |
| **Hospital type** |  |  |  |  |
| University, *n* = 1 286 | 111 (8.6) | 25 (1.9) | 277 (21.5) | 18 (6.5) |
| Centrum, *n* = 4 204 | 133 (3.2) | 153 (3.6) | 853 (20.3) | 118 (13.8) |
| Primary care, *n* = 2 165 | 26 (1.2) | 81 (3.7) | 292 (13.5) | 40 (13.7) |
| *p*-value | < 0.001 | 0.007 | < 0.001 | 0.004 |

**Table S10.2**. Emergency admission-related outcomes by stratification groups.

Values are *n* (%). Emergency admissions (*n* = 1053) followed by gastric cancer resection during the index hospitalization. *P*-values were determined using Chi-squared test.

|  | **Endoscopic resection** | **In-hospital mortality** | **Severe complications** | **Failure to rescue** |
| --- | --- | --- | --- | --- |
|  | ***n* = 50** | ***n* = 80** | ***n* = 393** | ***n* = 53** |
| **Annual resection volume** |  |  |  |  |
| >20, *n* = 89 | 11 (12.4) | 4 (4.5) | 36 (40.5) | 4 (11.1) |
| 14–20, *n* = 162 | 10 (6.2) | 8 (4.9) | 76 (46.9) | 6 (7.9) |
| 7–13, *n* = 362 | 18 (5.0) | 11 (8.6) | 131 (36.2) | 21 (16.0) |
| <7, *n* = 440 | 11 (2.5) | 37 (8.4) | 150 (34.1) | 22 (15.7) |
| *p*-value | < 0.001 | 0.291 | 0.031 | 0.375 |
| **Annual inpatient volume** |  |  |  |  |
| > 35 000, *n* = 1 916 | 24 (11.0) | 9 (4.1) | 91 (41.6) | 7 (7.7) |
| 9 000–35 000, *n* = 4 721 | 20 (3.5) | 59 (10.4) | 235 (41.3) | 40 (17.0) |
| < 9 000, *n* = 2 071 | 6 (2.3) | 12 (4.5) | 67 (25.3) | 6 (9.0) |
| *p*-value | < 0.001 | 0.001 | < 0.001 | 0.043 |
| **Hospital type** |  |  |  |  |
| University, *n* = 157 | 19 (12.1) | 4 (2.5) | 57 (36.3) | 4 (7.0) |
| Centrum, *n* = 551 | 22 (4.0) | 48 (8.7) | 235 (42.6) | 33 (14.0) |
| Primary care, *n* = 345 | 9 (2.6) | 28 (8.1) | 101 (29.3) | 16 (15.8) |
| *p*-value | < 0.001 | 0.033 | < 0.001 | 0.203 |
